# Poorer Quality of Life Outcomes Are Associated with Higher Levels of Stress, Lower Levels of Resilience, and Poorer Adjustment to Cancer in Outpatients Receiving Chemotherapy

**DOI:** 10.1016/j.soncn.2025.152015

**Published:** 2025-09-11

**Authors:** Jung Ae Kim, Astrid Block, Sueann Mark, Bruce A. Cooper, Steven M. Paul, Frances Cartwright, Marilyn J. Hammer, Yvette P. Conley, Jon D. Levine, Christine Miaskowski

**Affiliations:** a School of Nursing, University of California, San Francisco, California, USA; b Mount Sinai Medical Center, New York, New York, USA; c Phyllis F. Cantor Center, Dana Farber Cancer Institute, Boston, Massachusetts, USA; d School of Nursing, University of Pittsburgh, Pittsburgh, Pennsylvania, USA; e School of Medicine, University of California, San Francisco, California, USA

**Keywords:** Cancer, Chemotherapy, Comorbidity, Depression, Latent profile analysis, Mental adjustment to cancer, Patient-reported outcomes, Posttraumatic stress disorder, Quality of life, Resilience, Stress

## Abstract

**Objectives:**

The purposes were to identify subgroups of patients (n = 1324) with distinct quality of life (QOL) profiles and evaluate for differences among these subgroups in demographic and clinical characteristics, as well as levels of global, cancer-related, and cumulative life stress; resilience; and mental adjustment to cancer.

**Methods:**

Prior to their second or third cycle of chemotherapy, patients completed a demographic questionnaire, measures of stress (Perceived Stress Scale, global stress), Impact of Event Scale-Revised (IES-R, cancer-related distress), Life Stressor Checklist-Revised (LSC-R, cumulative life stress); resilience (Connor-Davidson Resilience Scale [CDRS]), and mental adjustment (Mental Adjustment to Cancer Scale [MAC]). In addition, they completed the Multidimensional QOL Scale – Patient Version six times over two cycles of chemotherapy. Latent profile analysis was used to identify the distinct QOL profiles. Parametric and nonparametric tests were used to evaluate for differences in risk factors among the QOL profiles.

**Results:**

Three distinct QOL profiles were identified (Low, 26.9%; Moderate, 44.7%; and High, 28.4%). Compared to the High QOL class, the other two classes were younger and more likely to be female and had a higher comorbidity burden and lower functional status. Differences among the QOL classes in PSS, IES-R, and LSC-R scores followed a similar pattern (Low > Moderate > High). Differences were found among the QOL classes in CDRS (Low < Moderate < High) and MAC (Low > Moderate > High) scores.

**Conclusions:**

This study is the first to describe interindividual variability in QOL outcomes among patients receiving chemotherapy. Levels of cancer-related stress reported by Low and Moderate QOL classes suggest that these patients meet the diagnostic criteria for posttraumatic stress and have levels of resilience below the normative score for the general population.

**Implications for Nursing Practice:**

Given that stress and resilience are modifiable risk factors, this information can be used by clinicians to design tailored interventions to improve patients’ QOL.

## INTRODUCTION

Quality of life (QOL) is one of the most important patient-reported outcomes (PROs) that require a need for ongoing evaluations during treatment. In fact, some patients choose to defer or forgo treatment if significant decrements in QOL may occur.^1^ Across various measures of QOL, multiple domains are evaluated (e.g., physical, psychological, social, and spiritual well-being).^2,3^ This type of evaluation provides clinicians with insights into patients’ treatment goals. It facilitates conversations between patients and clinicians that allow them to screen for and prioritize problems; engage in mutual decision-making regarding treatments; and monitor and manage the impact of treatments on patients’ QOL.^4^

As noted in one review,^5^ in addition to disease-free and overall survival, QOL outcomes are important metrics for patients, clinicians, and policy makers during an evaluation of the efficacy of various cancer treatments. Knowledge of how therapies impact QOL provides important information for all stakeholders in making treatment decisions. To that end, most clinical trials of new cancer treatments incorporate an evaluation of QOL. This work led to the publication of systematic reviews that summarized the impact of various treatments on the QOL of patients with cancer (e.g., see reviews on QOL outcomes in patients with esophageal cancer,^6^ prostate cancer,^7^ breast cancer,^8^ gynecologic cancer^9^). Findings regarding changes in QOL over time are variable given the diversity of cancer diagnoses, stages of disease, types of treatment, and duration of the study.

Of note, a large amount of interindividual variability exists in patients’ appraisal of their QOL during cancer treatment. Newer analytic techniques like latent variable modeling allow for the identification of subgroups of patients with distinct QOL profiles and subsequent evaluation of modifiable and nonmodifiable risk factors (e.g., demographic and clinical characteristics associated with poorer QOL outcomes). For example, in a study that evaluated women with breast cancer who were assessed prior to and for 6 months after surgery,^10^ two distinct profiles were identified (i.e., lower and higher QOL). Risk factors associated with membership in the lower QOL profile included: younger age, lower functional status, higher stage of disease, having an axillary lymph node dissection, and receipt of radiation and chemotherapy within the first 6 months following surgery. However, no studies were identified that evaluated interindividual variability in QOL in oncology outpatients receiving chemotherapy.

Cancer patients can experience heavy emotional tolls and psychological stress, especially when facing a life-threatening diagnosis that can be quite sudden and catastrophic.^11,12^ This stress can have a negative impact on patients’ overall QOL due to their fears of disease progression,^13^ concerns about side effects of chemotherapy,^14^ and worries about the future.^15^ In addition, chronic stress can have a lasting impact on patients’ QOL because prolonged activation of the sympathetic nervous system can stimulate inflammatory pathways,^16^ weaken immune responses,^17^ and increase the production of glucocorticoids, which can increase resistance to chemotherapy and influence tumor progression.^18^ However, most studies that evaluated the relationships between stress and QOL did not perform comprehensive assessments of different types of stress (i.e., global, disease-specific, and cumulative life stress).

Resilience plays a key role in enhancing the QOL of patients undergoing chemotherapy by helping them cope with the emotional and psychological challenges of treatment. Patients with higher levels of resilience report significantly better mental health outcomes,^19^ more effective use of strategies to cope with psychological distress,^20^ and lower levels of anxiety and depression.^21^ Resilient individuals are more likely to have a better experience during their treatment, as resilience serves as a protective factor, helping patients develop effective coping mechanisms and reduce their emotional distress.^22^ However, most of the studies that evaluated the relationship between resilience and QOL outcomes did not account for interindividual variability in patients’ appraisal of their QOL.

Mental adjustment is defined as how an individual mentally adapts to their diagnosis and treatment.^23^ This important psychological outcome impacts patients’ emotional health and QOL,^23^ as well as treatment outcomes.^24^ Improvements in mental adjustment to cancer are linked directly to decreases in anxiety and depression.^25^ Healthy mental adjustments, such as maintaining hope and having a sense of control, can create a greater fighting spirit, positive affect, and reduce feelings of fatalism, all of which contribute to a better QOL.^26,27^ In contrast, anxious preoccupation and cognitive avoidance, both of which are negative mental adjustments to illness, can decrease all domains of QOL.^28^ While improvements in mental adjustment to cancer are known to enhance emotional well-being and overall QOL, additional studies are needed that include an evaluation of associations between interindividual variability in QOL outcomes, stress, resilience, and mental adjustment to cancer in the same sample of patients.

Most of the studies cited above that reported on changes in the QOL of patients receiving chemotherapy used mean scores. However, an evaluation of the standard deviations reported in these papers suggests that a large amount of interindividual variability exists in patients’ appraisal of their QOL during chemotherapy. Equally important, little information is available on demographic, clinical, and stress characteristics associated with a worse QOL profile. Therefore, the purposes of this study, in a sample of patients undergoing chemotherapy (n = 1324), were to identify subgroups of patients with distinct QOL profiles and to evaluate for differences among these subgroups in demographic and clinical characteristics, as well as in measures of global, cancer-related, and cumulative life stress, resilience, and mental adjustment to cancer. An increased understanding of interindividual variability in this extremely important PRO and associated risk factors may allow clinicians to improve patients’ adherence to treatments and offer a more comprehensive approach to cancer care.^29^

## METHODS

### Patients and Settings

This longitudinal study is part of a larger study funded by the National Cancer Institute that evaluated patients’ symptom experience during chemotherapy.^30–33^ Eligible patients were ≥ 18 years of age; had a diagnosis of breast, gastrointestinal, gynecological, or lung cancer; had received chemotherapy within the preceding 4 weeks; were scheduled to receive at least two additional cycles of chemotherapy; were able to read, write, and understand English; and provided written informed consent. Patients were recruited from two Comprehensive Cancer Centers, one Veterans Affairs hospital, and four community-based oncology programs. A total of 2234 patients were approached, and 1343 consented to participate (60.1% response rate). The major reason for their refusal was being overwhelmed by their cancer treatment. A total of 1324 patients had complete data on the Multidimensional Quality of Life Scale–Patient Version (MQOLS-PV).^34^

### Instruments

#### Demographic and Clinical Characteristics

Patients completed a demographic questionnaire, the Karnofsky Performance Status (KPS) scale,^35^ the Alcohol Use Disorders Identification Test (AUDIT),^36^ and the Self-Administered Comorbidity Questionnaire (SCQ). The SCQ evaluates the occurrence of, treatments for, and impact of 13 common medical conditions.^37^ The MAX-2 score was used to evaluate the toxicity of the various chemotherapy regimens.^38^

#### QOL Measure

QOL was evaluated using the 41-item MQOLS-PV,^34^ which measures four dimensions of QOL (i.e., physical, psychological, social, and spiritual well-being scores) in cancer patients, as well as a total QOL score. Each item was rated on a 0-to-10 numeric rating scale (NRS) with higher scores indicating a better QOL.^2,34,39,40^ Cronbach’s alpha for the QOL-PV total score, which was used in the latent profile analysis (LPA), was 0.92.

#### Stress, Resilience, and Mental Adjustment to Cancer Measures

The 14-item Perceived Stress Scale (PSS) was used as a measure of global perceived stress according to the degree that life circumstances were appraised as stressful over the course of the previous week.^41^ Each item was rated on a 0-to-4 Likert scale (i.e., 0 = never, 1 = almost never, 2 = sometimes, 3 = fairly often, 4 = very often). In a probability sample drawn from the United States population,^42^ mean scores of 18.8 and 20.2 were reported by male and female participants, respectively. Total PSS scores can range from 0 to 56. Its Cronbach’s alpha was 0.89.

The 22-item Impact of Event Scale-Revised (IES-R) was used to measure cancer-related distress.^43,44^ Patients rated each item based on how distressing each potential difficulty was for them during the past week “with respect to their cancer and its treatment.” Each item was rated on a 0 (not at all) to 4 (extremely) Likert scale. Three sub-scales evaluate levels of intrusion, avoidance, and hyperarousal as perceived by the patient. The total IES-R score can range from 0 to 88. Sum scores of ≥24 indicate clinically meaningful posttraumatic symptomatology, and scores of ≥33 indicate probable posttraumatic stress disorder (PTSD).^45^ Cronbach’s alpha for the IES-R total score was 0.92.

The 30-item Life Stressor Checklist-Revised (LSC-R) is an index of lifetime trauma exposure (e.g., being mugged, sexual assault).^46^ The LSC − R assesses whether each stressful event occurred; at what ages the events occurred; how many times each event occurred; how dangerous the event was; and whether the individual had an intense emotional reaction to the event(s). The total LSC− R score is obtained by summing the total number of events endorsed (range of 0–30). If the patient endorsed an event, the patient was asked to indicate how much that stressor affected their life in the past year from 1 (not at all) to 5 (extremely). These responses were summed to yield a total “affected” sum score. In addition, a PTSD sum score was created based on the number of positively endorsed items (out of 21) that reflect the DSM-IV PTSD Criteria A for having experienced a traumatic event.

The 10-item Connor-Davidson Resilience Scale (CDRS) evaluates a patient’s personal ability to handle adversity (e.g., “I am able to adapt when changes occur”).^47,48^ Items are scored on a 5-point Likert scale (“not true at all” to “true nearly all of the time”). Total scores can range from 0 to 40, with higher scores indicating higher self-perceived resilience. The normative adult mean score in the United States is 31.8 (±5.4),^48,49^ with an estimated minimal clinically important difference of 2.7.^50^ Its Cronbach’s alpha was 0.90.

The 40-item Mental Adjustment to Cancer (MAC) Scale was designed to measure patients’ cognitive and behavioral responses to a cancer diagnosis and its treatment.^51^ It yields five subscales: fighting spirit (e.g., I am determined to beat this disease, 16 items), anxious preoccupation (e.g., I worry about the cancer returning or getting worse, 9 items), helpless/hopeless (e.g., I feel like giving up, 6 items), fatalism (e.g., I’ve had a good life, what’s left is a bonus, 8 items), and avoidance (e.g., I don’t really believe I have cancer, 1 item). Each item was rated on 1 (‘definitely does not apply to me’) to 4 (‘definitely applies to me’).^52–57^ Cronbach’s alphas for four of the five subscales were as follows: 0.81 for fighting spirit, 0.62 for anxious preoccupation, 0.82 for helpless/hopeless, and 0.53 for fatalism.

#### Study Procedures

This study was approved by the Committee on Human Research at the University of California, San Francisco, and by the institutional review board at each of the study sites. Written informed consent was obtained from all patients. Patients completed the MQOLS-PV, in their homes using paper questionnaires, a total of six times over two cycles of chemotherapy (i.e., prior to chemotherapy [Assessments 1 and 4], 1 week following the administration of chemotherapy [Assessments 2 and 5], and 2 weeks following the administration of chemotherapy [Assessments 3 and 6]). The remaining questionnaires were completed at enrollment (i.e., prior to the second or third cycle of chemotherapy). Medical records were reviewed for disease and treatment information.

#### Data Analysis

Using Mplus version 8.4,^58^ LPA was done to identify subgroups of patients with distinct QOL profiles. Latent profile analysis is a person-centered analytic technique that allows for the identification of unobserved subgroups (i.e., latent profiles) of individuals based on observed continuous variables. In this analysis, subgroups of patients with distinct QOL profiles were identified.^59–61^ Estimation was carried out with full information maximum likelihood with standard error and a χ^2^ test that are robust to nonnormality and nonindependence of observations (“estimator=MLR”). Model fit was evaluated to identify the solution that best characterized the observed latent class structure using the Bayesian Information Criterion, Vuong-Lo-Mendell-Rubin likelihood ratio test, entropy, and latent class percentages that were large enough to be reliable.^62^ Missing data were accommodated for with the use of the Expectation-Maximization algorithm.^63^

Using IBM SPSS Statistics version 29 (IBM Corporation, Armonk, NY), differences among the latent classes in demographic and clinical characteristics, as well as the stress, resilience, and mental adjustment to cancer measures were evaluated using analysis of variance, Kruskal-Wallis, or χ^2^ tests. A *P*-value of <.05 was considered statistically significant. Post hoc contrasts were done using a Bonferroni corrected *P*-value of <.017 (.05/3 possible pairwise comparisons).

## RESULTS

### Latent Class Solution

**Fig. 1.**
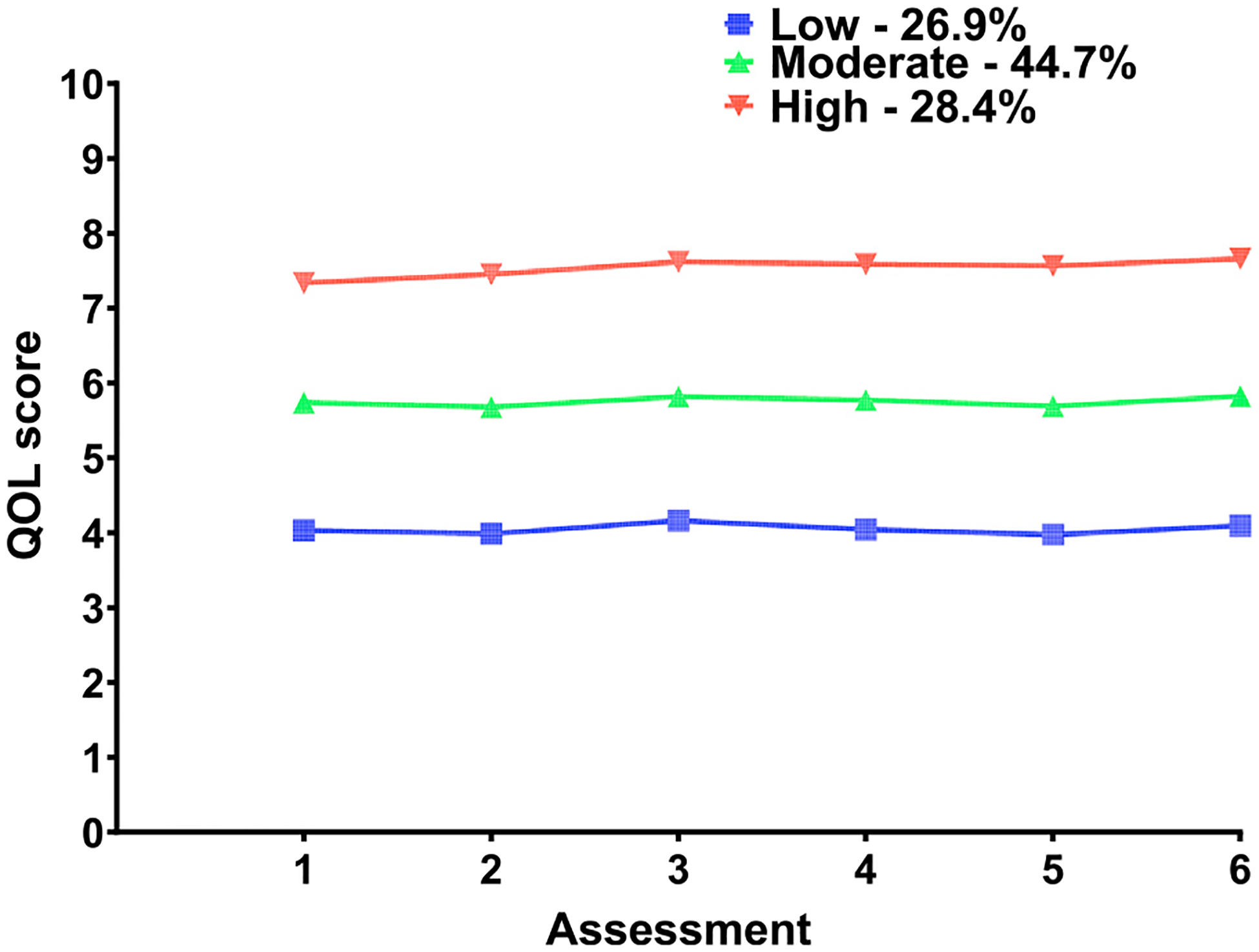
Trajectories of total quality of life scores for the three latent classes.

The rationale for the three-class solution is presented in Table 1. As shown in Fig. 1, of the 1324 patients in this study, 26.9% were in the Low, 44.7% in the Moderate, and 28.4% in the High QOL classes. For all three classes, the QOL scores remained relatively stable over time.

### Demographic and Clinical Characteristics

As shown in Table 2, age differences were found among the latent classes (Low < Moderate < High). Compared to the High class, patients in the other two classes were more likely to be female. Compared to the Moderate and High classes, the Low class was less likely to be married or partnered; more likely to self-report being Hispanic, mixed race, or other; less likely to be employed; more likely to self-report a lower annual income; and less likely to exercise on a regular basis.

In terms of clinical characteristics, differences among the three QOL classes in KPS (Low < Moderate < High) and SCQ scores and self-reported diagnosis of depression (Low > Moderate > High) followed a pattern. Compared to the other two classes, patients in the Low class were more likely to self-report a diagnosis of back pain and more likely to receive an antiemetic regimen containing a neurokinin-1 receptor antagonist and two other antiemetics. Compared to the High class, patients in the Low class had a higher MAX2 score and were more likely to have received surgery, chemotherapy, and radiation therapy.

### Stress, Resilience, and Mental Adjustment to Cancer

As shown in Table 3, differences among the three QOL classes in PSS scores; IES-R intrusion, avoidance, hyperarousal, and total scores; and all of the scores on the LSC-R followed a similar pattern (Low > Moderate > High). Significant differences were found among the three QOL classes in CDRS scores (Low < Moderate < High).

As shown in Table 4, differences among the classes in their scores for fighting spirit (Low < Moderate < High), as well as anxious preoccupation, helplessness/hopelessness, and fatalism (Low > Moderate > High) subscales of the MAC followed a pattern. Compared to the other two classes, patients in the Low class reported higher positive avoidance subscale scores.

## DISCUSSION

This study is the first to describe interindividual variability in QOL outcomes among outpatients receiving chemotherapy. The three distinct QOL profiles identified suggest that over 70% of the patients were experiencing moderate (44.7% in the Moderate class) to severe (26.9% in the Low class) decrements in their QOL. Equally important, these decrements in QOL persisted across the two cycles of chemo-therapy (Fig. 1).

While direct comparisons are not possible, in our previous study of patients with breast cancer (n = 397), who were assessed from prior to through 6 months after surgery using the same QOL measure,^10^ only two distinct QOL profiles were identified and named Lower (42.7%) and Higher (57.3%) QOL. For both profiles, the QOL scores remained relatively consistent over time. However, for the patients with breast cancer, the Lower QOL classes’ scores were equivalent to the QOL scores of the Moderate class in the current study (i.e., ~ 6 on a 0-to-10 scale). Therefore, it is important to note that over 25% of the current sample had very low QOL scores (i.e., ~ 4 on a 0-to-10 scale). Any number of factors may explain the differences in the total number of classes identified in these two samples, including differences in sample sizes, evaluation of patients with heterogeneous versus homogeneous (i.e., only breast cancer) types of cancer, differences in cancer treatments, and differences in the timing of the assessments. However, findings across both studies suggest that when interindividual differences in QOL are evaluated using a person-centered analytic approach, the within-class decrements in QOL remain relatively stable. This hypothesis warrants confirmation in future longitudinal studies of QOL outcomes, particularly in patients receiving chemotherapy and other types of systemic treatment. The remainder of the discussion highlights modifiable and nonmodifiable risk factors associated with decrements in QOL (Table 5).

### Demographics and Clinical Characteristics

The common demographic and clinical characteristics associated with membership in the Moderate and Low QOL classes were younger age, being female, having a lower functional status and a higher comorbidity burden, being more likely to self-report a diagnosis of depression, and being more likely to have received one or more prior cancer treatments. In terms of age^10,64^ and gender,^65^ our findings are consistent with previous reports. One can hypothesize that younger patients may find it more difficult to deal with the significant challenges and life changes (e.g., career or family obligations) imposed by cancer and its treatments. These interruptions in life may lead to increases in stress and associated decrements in QOL. Female patients may face gender-specific challenges (e.g., changes in family caregiving roles, concerns about fertility) that lead to increased stress.

While consistent with previous reports of associations between functional status^10,66^ and multimorbidity^10,66,67^ and QOL outcomes, differences among the classes in both clinical characteristics exhibited a dose-response effect. That is, as functional status worsened and the comorbidity burden increased, the overall QOL decreased. In fact, the differences in KPS scores between the High class and the Moderate (Cohen’s d = 0.5) and Low (Cohen’s d = 1.1) classes, respectively, represent not only statistically significant but also clinically meaningful decrements in functional status.^68–70^ Equally important, the KPS score of 73.1 reported by the Low class is characterized by being able to care for oneself, but unable to carry on normal activity or do active work.^35^ Additional research is warranted on the longitudinal relationships between changes in functional status and QOL in patients undergoing chemotherapy.

Multimorbidity is defined as an individual having two or more chronic conditions.^71,72^ While all of the classes mean number of comorbid conditions exceeded two, and for the Low class, the number of comorbidities on the SCQ was as high as 11 of 13. In terms of specific comorbid conditions, depression was the only comorbid condition that had higher prevalence rates in both the Low (40.4%) and Moderate (15.7%) classes compared to the High (4.8%) class. In a recent review,^73^ the global prevalence rate for depression in patients with cancer was 33.2%. The key risk factors for depression include advanced-stage disease, poor social support, and inadequate sleep.

An association between depressive symptoms and poorer QOL outcomes may be partially explained by the fact that some patients are not able to adapt to various stressors and experience feelings of pessimism.^74^ In addition, depression is associated with decreased adherence with treatments^75^ and lower levels of physical activity^76^ that can result in decrements in QOL. Equally important, depression contributes to the development of a number of chronic conditions (e.g., hypertension, diabetes) and associated decrements in physical function.^77^ Clinicians need to monitor for depression in patients with cancer and provide referrals to psychological support services.

As noted in Table 5, being of Hispanic, mixed race, or other racial/ethnic group; being unmarried or partnered, living alone, being unemployed, having a lower annual household income, and reporting a lack of regular exercise were unique risk factors associated with membership in the Low QOL class. Collectively, these demographic characteristics represent several important social determinants of health.^78^ One can hypothesize that patients belonging to a minority racial or ethnic group may not have the same access to and/or financial resources to receive optimal symptom management with resultant decrements in QOL. In addition, they may have language barriers that decrease their ability to understand instructions for self-management of their cancer treatments.

Equally important, being unemployed is associated with decrements in QOL because of a lack of financial security, lower physical and functional well-being, and lower self-esteem.^79,80^ In addition, as noted in one review,^81^ oncology patients who experience financial toxicity have increased difficulty managing their symptoms and significant decrements in their ability to work. In addition, these patients may not have the time to exercise, which results in increases in depressive symptoms and decrements in functional status.^82,83^

A higher MAX 2 score, the occurrence of back pain, the prior receipt of multiple cancer treatments, and the receipt of a potent antiemetic regimen were the unique clinical risk factors associated with membership in the Low QOL class. The higher MAX2 score and the receipt of a potent antiemetic regimen suggest that these patients received more toxic chemotherapy regimens. Both of these characteristics can result in a higher symptom burden and physical impairments that contribute to decrements in QOL. In terms of the occurrence of back pain, compared to the High (18.4%) class, 38.2% of the patients in the Low class reported back pain. This percentage is similar to the 39.0% prevalence rate for this chronic condition among adults in the United States.^84^ The disability associated with back pain can have a negative impact on all aspects of a patient’s QOL.^85,86^ Given that patients can experience cancer (e.g., chemotherapy-induced neuropathy, spinal cord compression, joint pain, muscle aches) and/or noncancer (e.g., headache, osteoarthritis) pain from multiple causes, additional research is warranted on the impact of various types of pain on all of the domains of QOL.

Equally important, patients in the Low QOL class were more likely to have received a combination of surgery, radiation therapy, and chemotherapy, prior to their current cancer treatment. These findings are consistent with prior work that demonstrated that receipt of multiple cancer treatments is associated with a higher symptom burden,^87,88^ changes in body composition (e.g., muscle wasting),^89 90^ and overall decrements in physical functioning. All of the negative consequences of multiple types of cancer treatment can result in significant decrements in QOL.

### Stress, Resilience, and Mental Adjustment to Cancer

It is interesting to note that for all of the stress measures, a “dose-response effect” was observed. While one cannot determine causality, these findings suggest strong interrelationships among higher levels of global, cancer-specific, and cumulative life stress and decrements in patients’ overall QOL. In terms of global stress, the Low class had PSS scores (i.e., 25.8) that were higher than the scores of 18.8 to 20.2 reported by a sample of males and females, respectively, who were drawn from the general population of the United States.^42^ One advantage of using the IES-R is that it has cutoff scores for clinically meaningful levels of PTSD symptomatology and probable PTSD.^45^ In terms of PTSD symptomatology, 26.4% of the patients in the Moderate and 58.3% of the patients in the Low QOL classes reported scores above the cutoff. For probable PTSD, the percentages were 9.1% and 35.7% for the Moderate and Low classes, respectively. While the rates of PTSD in the Moderate class are consistent with the 17%^91^ and 44%,^92^ found in previous studies, the rates in the Low class were higher. Additional research is needed to determine differences in specific life stressors reported by patients in the three QOL classes that contribute to the development of PTSD symptomatology.

Our findings are consistent with previous reports that found that elevated levels of stress have a negative impact on patients’ psychosocial adjustment to cancer and are associated with poorer physical and mental health during treatment.^93,94^ One can hypothesize that higher levels of stress can make it more difficult for patients to accept their diagnosis, engage in their treatment regimen, and/or maintain hope. Equally important, the trauma of a cancer diagnosis can cause uncertainty and loss of control over one’s life.^94,95^ In addition, chronic stress can result in changes in the hypothalamic-pituitary-adrenal axis that can decrease immune function and contribute to worse outcomes.^96^ Stress, which can include concerns about prognosis, treatment efficacy, and life disruptions, can affect QOL throughout treatment. Over time, QOL can deteriorate due to the mental burden placed on the patient, as well as increases in symptom burden, and a general decline in one’s ability to cope. In fact, evidence suggests that interventions like mindfulness training, which specifically target stress, result in improvements in QOL outcomes.^97,98^ These types of interventions help patients to regain a sense of control and reduce feelings of anxiety. In addition, mindfulness interventions may facilitate increases in resilience.^99,100^

Differences in levels of resilience among the QOL classes, defined as an individual’s ability to adapt to stressful situations,^101^ exhibited a dose-response effect. Of note, patients in both the Low and Moderate QOL classes had CDRS scores that were below the mean score for the US general population. These low scores suggest that patients are struggling to cope with the burden of their cancer diagnosis and are experiencing high levels of distress, feelings of helplessness, and a decreased sense of control.

Higher levels of resilience help to promote recovery, restore a purpose in one’s life,^102^ and are associated with better QOL outcomes.^103,104^ One can hypothesize that resilient individuals are more likely to seek support, socialize, be engaged in their care, and maintain hope. In addition, individuals with higher levels of resilience may be more confident in making treatment decisions and advocating for themselves, which results in increased adherence and positive treatment outcomes. Equally important, resiliency is associated with better physical and mental health because these patients can self-regulate their emotions and show more self-compassion towards themselves.^105^ The development of self-care strategies can help patients to deal with their emotions and improve their QOL.^105–107^ Furthermore, fostering resilience can play a critical role in patients’ mental adjustment to cancer, as higher levels of resilience allow them to process and adapt to significant changes in their lives.^105,108^

Similar to stress and resilience, except for the positive avoidance subscale, all of the other MAC subscale scores exhibited a dose response effect. However, patients in the Low QOL class had the lowest score for fighting spirit and the highest scores for anxious preoccupation, helplessness/hopelessness, fatalism, and positive avoidance. These findings are consistent with previous reports that found significant correlations between mental adjustment and QOL scores and suggest that patients’ ability to adjust to their cancer and its treatments influences their overall well-being and health outcomes.^23,27,28^ Effective adjustment to cancer leads to a positive effect, which helps to broaden patients’ perspectives and assists them in developing effective problem-solving coping strategies, with associated improvements in health outcomes.^27^ Successful mental adjustment can help patients feel that they have a sense of control over their lives, are able to reestablish their self-esteem, and adopt a positive attitude.^23^ On the other hand, maladaptive adjustments, such as feelings of helplessness and hopelessness, can cause significant decreases in emotional and functional well-being. Patients may be less inclined to participate in social activities and care for their health.^28^ While the extant literature suggests that mental adjustment is crucial for improving QOL, additional research is needed to fully explore the relationships among stress, resilience, and mental adjustment to cancer and various domains of QOL.

### Limitations

Several limitations warrant consideration. Given that changes in QOL were assessed for only a 2-month period, additional longitudinal studies are needed to obtain a more comprehensive evaluation of whether these QOL profiles persist following the completion of treatment and into survivorship. Additional research is warranted to determine the optimal timing of QOL assessments to be able to confirm the most vulnerable times when decrements in QOL occur. Of note, the large sample of patients was relatively homogenous in terms of age, gender, education, and ethnicity. Therefore, these findings may not generalize to patients from more diverse ethnic and socioeconomic backgrounds. In addition, because the most frequently cited reason patients declined participation was because they were overwhelmed with cancer treatments, the findings may underestimate the impact of chemotherapy on patients’ level of stress, resilience, and mental adjustment to cancer and QOL outcomes

### Implications for Clinical Practice and Research

Despite these limitations, findings from this study provide new insights on the modifiable and nonmodifiable risk factors associated with significant decrements in QOL. Based on the resources available in each clinical setting, clinicians can use this information to identify patients who are at increased risk for poorer QOL outcomes. A focus should be placed on targeting interventions for specific modifiable risk factors. For example, patients who report a poorer functional status and lack of regular exercise can be referred to physical therapy for a tailored evaluation and activity plan. In addition, oncology clinicians can consult with primary care providers to optimize the management of multiple comorbid conditions (e.g., depression, back pain).

Given the significant findings related to stress, resilience, and mental adjustment to cancer, clinicians can refer patients for a variety of nonpharmacologic interventions (e.g., mindfulness-based stress reduction^109–111^) to improve their QOL. Additional longitudinal research is warranted to evaluate QOL trajectories across diverse populations; assess the impact of targeted interventions to improve QOL; and examine relationships among other characteristics (e.g., hope, specific adverse childhood experiences, severity of common symptoms) and interindividual variability in multiple domains of QOL.

## Figures and Tables

**TABLE 1:** Latent Profile Solutions and Fit Indices for One through Four Classes for the Total Quality of Life Scores

Model	LL	AIC	BIC	Entropy	VLMR
1 Class	−8094.41	16,230.81	16,339.77	n/a	n/a
2 Class	−7612.02	15,280.04	15,425.32	0.77	964.77^‡^
3 Class^a^	−7332.52	14,735.05	14,916.64	0.80	558.99^†^
4 Class	−7143.38	14,370.76	14,588.67	0.83	ns

Baseline entropy and VLMR are not applicable to the one-class solution.

† p < 0.01.

‡ p < 0.00005.

a The three-class solution was selected because the BIC for that solution was lower than the BIC for the 2-class solution. In addition, the VLMR was significant for the three-class solution, indicating that three classes fit the data better than two classes. Although the BIC was smaller for the four-class than for the three-class solution, the VLMR was not significant for the 4-class solution, indicating that too many classes were extracted.

Abbreviations: AIC, Akaike’s Information Criterion; BIC, Bayesian Information Criterion; LL, log-likelihood; n/a, not applicable; ns, not significant; VLMR, Vuong-Lo-Mendell-Rubin likelihood ratio test for the K v. K-1 model.

**TABLE 2:** Differences in Demographic and Clinical Characteristics Among the Quality of Life Classes at Enrollment

Characteristic	Low (1) 26.9% (n=356) Mean (SD)	Moderate (2) 44.7% (n=592) Mean (SD)	High (3) 28.4% (n=376) Mean (SD)	Statistics
Age (years)	54.6 (12.3)	56.7 (12.6)	60.1 (11.3)	F = 19.78, *P* < .001 1 < 2 < 3
Education (years)	16.0 (3.0)	16.3 (3.0)	16.2 (3.0)	F = 0.93, *P* = .395
Body mass index (kg/m^2^)	26.5 (6.2)	26.0 (5.5)	26.2 (5.3)	F = 0.86, *P* = .424
Alcohol Use Disorders Identification Test score	3.0 (2.7)	3.1 (2.5)	2.8 (2.1)	F = 0.93, *P* = .394
Karnofsky Performance Status score	73.1 (11.3)	80.1 (12.0)	86.5 (10.8)	F = 119.78, *P* < .001 1 < 2 < 3
Number of comorbidities out of 13	2.9 (1.5)	2.3 (1.3)	2.2 (1.4)	F = 26.19, *P* < .001 1 > 2 and 3
Self-administered Comorbidity Questionnaire score	6.7 (3.6)	5.3 (2.9)	4.6 (2.9)	F = 42.41, *P* < .001 1 > 2 > 3
Time since diagnosis (years)	2.2 (4.4)	2.0 (3.9)	1.7 (3.2)	KW = 5.48, *P* = 0.65
Time since diagnosis (median)	0.45	0.42	0.40	
Number of prior cancer treatments	1.7 (1.5)	1.6 (1.5)	1.5 (1.4)	F = 2.62, *P* = .073
Number of metastatic sites including lymph nodes	1.3 (1.3)	1.3 (1.3)	1.2 (1.1)	F = 0.58, *P* = .560
Number of nonlymph metastatic sites	0.8 (1.1)	0.8 (1.1)	0.7 (0.9)	F = 0.96, *P* = .383
MAX2 score	0.18 (0.08)	0.18 (0.08)	0.16 (0.08)	F = 3.45, *P* = .032 1 > 3
	% (n)	% (n)	%(n)	
Female	83.7 (298)	79.2 (468)	70.5 (265)	*χ*^2^ = 19.59, *P* <.001 1 and 2 > 3
Self-reported race/ethnicity				*χ*^2^ = 22.75, *P* < .001
White	65.8 (231)	73.0 (428)	67.4 (250)	NS
Asian or Pacific Islander	10.8 (38)	12.8 (75)	13.5 (50)	NS
Black	6.6 (23)	6.7 (39)	8.9 (33)	NS
Hispanic, Mixed, or Other	16.8 (59)	7.5 (44)	10.2 (38)	1 > 2 and 3
Married or partnered (% yes)	55.6 (195)	66.2 (385)	70.2 (261)	*χ*^2^ = 18.15, *P* < .001 1 < 2 and 3
Lives alone (% yes)	27.1 (95)	21.4 (125)	16.7 (62)	*χ*^2^ = 11.71, *P* = .003 1 > 3
Currently employed (% yes)	23.4 (83)	36.6 (215)	43.5 (161)	*χ*^2^ = 33.24, *P* < .001 1 < 2 and 3
Annual household income				KW = 19.77, *P* < .001
Less than $30,000^+^	27.0 (88)	16.7 (89)	12.6 (41)	1 < 2 and 3
$30,000 to $70,000	23.3 (76)	20.6 (110)	20.3 (66)	
$70,000 to $100,000	12.3 (40)	17.6 (94)	20.3 (66)	
Greater than $100,000	37.4 (122)	45.1 (241)	46.8 (152)	
Childcare responsibilities (%yes)	25.4 (88)	22.0 (127)	19.7 (73)	*χ*^2^ = 3.33, *P* = .190
Elder care responsibilities (% yes)	10.5 (34)	7.7 (41)	5.8 (20)	*χ*^2^ = 5.15, *P* = .076
Past or current history of smoking (% yes)	35.9 (126)	36.6 (213)	32.5 (120)	*χ*^2^ = 1.73, *P* = .421
Exercise on a regular basis (% yes)	64.0 (220)	71.6 (414)	76.6 (285)	*χ*^2^ = 14.10, *P* < .001 1 < 2 and 3
Specific comorbid conditions (% yes)				
Heart disease	5.9 (21)	5.6 (33)	4.8 (18)	*χ*^2^ = 0.48, *P* = .787
High blood pressure	28.9 (103)	29.2 (173)	32.7 (123)	*χ*^2^ = 1.67, *P* = .435
Lung disease	15.2 (54)	9.3 (55)	10.6 (40)	*χ*^2^ = 7.89, *P* = .019 1 > 2
Diabetes	8.7 (31)	8.8 (52)	9.3 (35)	*χ*^2^ = 0.10, *P* = .950
Ulcer or stomach disease	7.3 (26)	4.1 (24)	4.0 (15)	*χ*^2^ = 5.98, *P* = .050
Kidney disease	2.2 (8)	1.2 (7)	1.1 (4)	*χ*^2^ = 2.29, *P* = .318
Liver disease	5.3 (19)	7.1 (42)	6.4 (24)	*χ*^2^ = 1.14, *P* = .564
Anemia or blood disease	15.4 (55)	12.2 (72)	9.6 (36)	*χ*^2^ = 5.87, *P* = .053
Depression	40.4 (144)	15.7 (93)	4.8 (18)	*χ*^2^ = 158.24, *P* < .001 1 > 2 > 3
Osteoarthritis	14.6 (52)	11.0 (65)	10.9 (41)	*χ*^2^ = 3.31, *P* = .191
Back pain	38.2 (136)	22.6 (134)	18.4 (69)	*χ*^2^ = 42.79, *P* < .001 1 > 2 and 3
Rheumatoid arthritis	3.9 (14)	2.4 (14)	3.5 (13)	*χ*^2^ = 2.05, *P* = .335
Type of cancer				*χ*^2^ = 14.62, *P* = .023
Breast	43.8 (156)	39.5 (234)	38.8 (146)	NS
Gastrointestinal	27.2 (97)	30.4 (180)	33.5 (126)	NS
Gynecologic	17.4 (62)	20.1 (119)	13.0 (49)	2 > 3
Lung	11.5 (41)	10.0 (59)	14.6 (55)	NS
Type of prior cancer treatment				*χ*^2^ = 17.39, *P* = .008
No prior treatment	21.9 (77)	23.3 (133)	30.5 (112)	1 and 2 < 3
Only surgery, CTX, or RT	42.6 (150)	44.1 (252)	37.9 (139)	NS
Surgery and CTX, or surgery and RT, or CTX and RT	17.9 (63)	20.1 (115)	21.3 (78)	NS
Surgery and CTX and RT	17.6 (62)	12.4 (71)	10.4 (38)	1 > 3
Metastatic sites				*χ*^2^ = 4.98, *P* = .29
No metastasis	33.6 (118)	31.9 (186)	32.1 (120)	
Only lymph nodes	21.7 (76)	22.0 (128)	22.5 (84)	
Only nonlymph nodes	19.4 (68)	22.1 (129)	21.1 (79)	
Lymph nodes and other sites	25.4 (89)	24.0 (140)	24.3 (91)	
Chemotherapy regimen				*χ*^2^ = 4.98, *P* = .29
Only CTX	72.1 (251)	71.5 (414)	65.5 (243)	
Only targeted therapy	2.9 (10)	2.8 (16)	3.5 (13)	
Both CTX and targeted therapy	25 (87)	25.7 (149)	31 (115)	
Cycle length				KW = 0.34, *P* = .343
14-day cycle	40.5 (142)	42.5 (249)	41.9 (157)	
21-day cycle	52.1 (183)	50.3 (295)	50.9 (191)	
28-day cycle	7.4 (26)	7.2 (42)	7.2 (27)	
Emetogenicity of the CTX regimen				KW = 0.02, *P* = .988
Minimal/low	21.9 (77)	19.3 (113)	17.9 (67)	
Moderate	56.4 (198)	61.8 (363)	64 (240)	
High	21.7 (76)	18.9 (111)	18.1 (68)	
Antiemetic regimen				*χ*^2^ = 20.83, *P* = .002
None	6.8 (23)	7.5 (43)	7.0 (26)	NS
Steroid alone or serotonin receptor antagonist alone	21.2 (72)	19.2 (110)	22.5 (83)	NS
Serotonin receptor antagonist and steroid	39.4 (134)	49.9 (286)	50.9 (188)	1 < 2 and 3
NK-1 receptor antagonist and two other antiemetics	32.6 (111)	23.4 (134)	19.5 (72)	1 > 2 and 3

a Total number of metastatic sites evaluated was 9.

+ Reference group

Abbreviations: CTX, chemotherapy; KW, Kruskal-Wallis; NK-1, neurokinin-1; NS, not significant; RT, radiation therapy; SD, standard deviation.

**TABLE 3:** Differences in Stress and Resilience Scores Among the Quality of Life Classes at Enrollment

Measures	Low (1) 26.9% (n = 356) Mean (SD)	Moderate (2) 44.7% (n = 592) Mean (SD)	High (3) 28.4% (n = 376) Mean (SD)	Statistics
PSS total score (range 0 to 56)	25.8 (7.5)	18.2 (6.2)	12.2 (5.7)	F = 403.33, *P* < .001 1 > 2 > 3
IES-R total score	29.0 (14.9)	18.0 (10.3)	10.6 (8.1)	F = 237.30, *P* < .001 1 > 2 > 3
≥24 - clinically meaningful PTSD symptomatology				
≥33 - probable PTSD				
IES-R intrusion	1.5 (0.8)	0.9 (0.6)	0.5 (0.4)	F = 247.32, *P* < .001 1 > 2 > 3
IES-R avoidance	1.2 (0.8)	1.0 (0.6)	0.7 (0.6)	F = 70.35, *P* < .001 1 > 2 > 3
IES-R hyperarousal	1.2 (0.8)	0.6 (0.5)	0.3 (0.3)	F = 251.14, *P* < .001 1 > 2 > 3
LSC-R total score (range 0 to 30)	7.3 (4.6)	5.9 (3.8)	5.2 (3.3)	F = 25.53, *P* < .001 1 > 2 > 3
LSC-R affected sum (range 0 to 150)	17.2 (14.5)	11.0 (9.0)	8.5 (7.5)	F = 54.05, *P* < .001 1 > 2 > 3
LSC-R PTSD sum (range 0 to 21)	4.2 (3.5)	3.0 (3.0)	2.2 (2.2)	F = 34.72, *P* < .001 1 > 2 > 3
CDRS total score (31.8 (±5.4) is the normative score for the general population of the United States)	26.3 (6.8)	30.1 (5.6)	33.6 (5.0)	F = 142.48, *P* < .001 1 < 2 < 3

Abbreviations: CDRS = Connor Davidson Resilience Scale, IES-R = Impact of Event Scale - Revised, LSC-R = Life Stressor Checklist-Revised, PSS = Perceived Stress Scale, PTSD = posttraumatic stress disorder, SD = standard deviation

a Clinically meaningful cutoff scores or range of scores

**TABLE 4:** Differences in Mental Adjustment to Cancer Subscale Scores Among the Quality of Life Classes at Enrollment

Subscales	Low (1) 26.9% (n = 356) Mean (SD)	Moderate (2) 44.7% (n = 592) Mean (SD)	High (3) 28.4% (n = 376) Mean (SD)	Statistics
Fighting spirit	48.3 (5.7)	51.7 (5.1)	55.0 (4.9)	F = 144.72, *P* < .001 1 < 2 < 3
Anxious preoccupation	26.0 (3.3)	23.1 (3.5)	21.3 (3.1)	F = 182.19, *P* < .001 1 > 2 > 3
Helpless, hopelessness	11.5 (3.0)	8.7 (2.5)	7.3 (1.8)	F = 263.66, *P* < .001 1 > 2 > 3
Fatalism	18.5 (3.3)	17.6 (3.2)	16.6 (3.5)	F = 28.46, *P* < .001 1 > 2 > 3
Positive avoidance	1.7 (0.8)	1.5 (0.7)	1.5 (0.8)	F = 9.27, *P* < .001 1 > 2 and 3

Abbreviation: SD, standard deviation.

**TABLE 5:** Characteristics Associated with Membership in the Low and Moderate Quality of Life Classes Compared to the High Quality of Life Class

Characteristic^a^	Moderate quality of life class	Low quality of life class
Demographic Characteristics		
More likely to be younger	■	■
More likely to be female	■	■
More likely to be Hispanic, mixed race, or other		■
Less likely to be married or partnered		■
More likely to live alone		■
Less likely to be currently employed		■
More likely to have a lower annual household income		■
Less likely to exercise on a regular basis		■
Clinical Characteristics		
Lower functional status (Karnofsky Performance Status score)	■	■
Higher number of comorbid conditions		■
Higher comorbidity burden (Self-administered Comorbidity Questionnaire)	■	■
Higher MAX2 score		■
More likely to self-report depression	■	■
More likely to self-report back pain		■
More likely to have gynecologic cancer	■	
Less likely to have received no prior cancer treatment	■	■
More likely to have received surgery, chemotherapy, and radiation therapy		■
Less likely to have received a serotonin receptor antagonist and a steroid		■
More likely to have received a neurokinin-1 receptor antagonist and two other antiemetics		■
Stress and Resilience Characteristics		
Higher Perceived Stress Scale total score	■	■
Higher Impact of Event Scale-Revised total score	■	■
Higher Impact of Event Scale-Revised intrusion subscale score	■	■
Higher Impact of Event Scale-Revised avoidance subscale score	■	■
Higher Impact of Event Scale-Revised IES-R hyperarousal subscale score	■	■
Higher Life Stressor Checklist-Revised total score	■	■
Higher Life Stressor Checklist-Revised affected sum score	■	■
Higher Life Stressor Checklist-Revised Post Traumatic Stress Disorder sum score	■	■
Lower Connor Davidson Resilience Scale total score	■	■
Mental Adjustment to Cancer Characteristics		
Lower fighting spirit subscale score	■	■
Higher anxious preoccupation subscale score	■	■
Higher helpless, hopelessness subscale score	■	■
Higher fatalism subscale score	■	■
Higher positive avoidance subscale score		■

a Comparisons done with the High Quality of Life Class

■ - Indicates the presence of the risk factor compared to the High Quality of Life Class

## Layperson Summary

### What we investigated and why

Quality of life is one of the most important patient-reported outcomes. Very little information is available on the relationships among stress, resilience, and how patients adjust to their cancer and their appraisal of their quality of life.

### How we did our research

Patients who were receiving chemotherapy were asked to complete questionnaires that evaluated their levels of stress, resilience, and mental adjustment to cancer as well as their overall quality of life. Using a novel statistical approach, patients were grouped based on their quality of life scores over two cycles of chemotherapy.

### What we found

Three distinct quality of life profiles were found. Patients with higher levels of stress, lower levels of resilience, and poorer adjustment to cancer reported the lowest quality of life scores.

### What it means

Clinicians need to assess stress levels in patients receiving chemotherapy and make referrals to psychological services to assist to reduce stress and improve their levels of resilience. This intervention may result in significant improvements in patients’ quality of life.
